# Evaluation of synergistic effect of tazobactam with meropenem and ciprofloxacin against multi-drug resistant Acinetobacter baumannii isolated from burn patients in Tehran

**DOI:** 10.3205/dgkh000324

**Published:** 2019-08-02

**Authors:** Sahel Valadan Tahbaz, Leila Azimi, Mahla Asadian, Abdolaziz Rastegar Lari

**Affiliations:** 1 Department of Microbiology, Islamic Azad University, North Tehran Branch, Tehran, Iran; 2 Pediatric Infections Research Center, Research Institute for Children’s Health, Shahid Beheshti University of Medical Sciences, Tehran, Iran; 3 Division of Microbiology, Department of Pathobiology, School of Public Health, Tehran University of Medical Sciences, Tehran, Iran; 4 Department of Microbiology, Iran University of Medical Sciences, Tehran, Iran

**Keywords:** Acinetobacter baumannii, tazobactam, MIC, carbapenem resistance

## Abstract

**Background:** *Acinetobacter baumannii* is an increasingly important cause of nosocomial infections worldwide. In addition to the intrinsic resistance of *Acinetobacter baumannii* to many antibiotics, available treatment approaches with older antibiotics are significantly associated with an increase in multiresistant strains. The aim of this study was to evaluate the synergistic effect of tazobactam with meropenem and ciprofloxacin against carbapenems and drug resistant *Acinetobacter baumannii* isolated from burn patients in a tertiary burn center in Tehran.

**Materials and methods:** In this study, a total of 47 clinical isolates of *A. baumannii* were included from burn patients admitted to the Shahid Motahari Burns Hospital, Tehran, from June 2018 to August 2018. The disk diffusion method was used to determine resistance patterns. The synergistic effect of tazobactam with meropenem and ciprofloxacin was evaluated by determining the MIC. A PCR assay was performed to determine *bla_OXA-40-like_*, *bla_OXA-58-like_* and *bla_OXA-24-like_*.

**Results:** Antibiotic susceptibility testing revealed that all of the isolates were resistant to meropenem and ciprofloxacin. The MIC values decreased in the cases of combined use of ciprofloxacin and meropenem with tazobactam. The bla_OXA-24-like_ gene was the predominant carbapenemase gene (93.6%), followed by *bla_OXA-40-like_*, which was detected in 48.9% of isolates. None of the *A. baumannii* isolates harbored the *bla_OXA-58-like_* gene.

**Conclusions:** Based on in-vitro antimicrobial susceptibility in the current study, the MIC of tazobactam combined with meropenem or ciprofloxacin have been shown to be variable. Furthermore, the data acquired from such *in vitro* conditions should be confirmed by reliable results from sufficiently controlled clinical trials.

## Zusammenfassung

**Hintergrund:** Nosokomiale Infektionen durch *Acinetobacter baumannii* nehmen weltweit an Bedeutung zu und sind zunehmend Ursache schwer beherrschbarer Ausbrüche. Neben der intrinsischen Resistenz von *A. baumannii* gegenüber vielen Antibiotika ist eine dramatische Zunahme multiresistenter Stämme fast immer durch Carbapenemasen bedingt zu verzeichnen. Daher sollte die Kombination von Tazobactam mit Meropenem und Ciprofloxacin auf synergistische Wirkung gegen multiresistente *A. baumannii* Stämme, isoliert von Verbrennungspatienten des Verbrennungszentrums Shahid Motahari in Teheran, geprüft werden.

**Methode:** Im Zeitraum Juni bis August 2018 wurden 47 klinische Isolate von *A. baumannii* mit Hilfe des Plättchen Diffusionstests auf Synergismus von Tazobactam mit Meropenem und Ciprofloxacin geprüft. Die Carbapenemasegene *bla_OXA-40-like_*, *bla_OXA-58-like_* and *bla_OXA-24-like_* wurden mittels PCR bestimmt.

**Ergebnisse:** Alle Isolate erwiesen sich als resistent gegen Meropenem and Ciprofloxacin. Die minimale Hemmkonzentration (MHK) wurde in der Kombination von Ciprofloxacin und Meropenem mit Tazobactam reduziert. Unter der Carbapenemasegenen dominierte das *bla_OXA-24-like_* mit 93,6%, gefolgt von *bla_OXA-40-like_*, das in 48,9% der Isolate nachgewiesen wurden, während kein Isolat das *bla_OXA-58-like_* Gen enthielt.

**Schlussfolgerung:** Die MHK von Tazobactam in Kombination mit Meropenem oder Ciprofloxacin verhielt sich unterschiedlich. In jedem Fall müssen derartige in vitro generierte Ergebnisse in kontrollierten klinischen Studien bestätigt werden.

## Background

Burn-wound infections are considered as one of the important causes of death in developing countries [1]. Patients with severe burns are at high risk of acquiring nosocomial pathogens and contracting numerous infections as a result of the immunocompromising effects of burns, cutaneous and respiratory tract injury, prolonged hospital stays, and invasive diagnostic methods and treatment procedures [2], [3]. The control and prevention of life-threatening infectious diseases among burn patients remains a major concern worldwide, as the environment in burn units can become contaminated with resistant opportunistic pathogens [3].

*Acinetobacter baumannii* is considered an important nosocomially acquired opportunistic pathogen causing a wide range of severe infections, including those of burn-wounds, surgical wounds, the urinary tract (UTI), ventilator-associated pneumonia (VAP), as well as nosocomial meningitis and bacteremia [4], [5]. The bacterium is highly successful in persisting and spreading in the hospital environment, and thus can survive under dry, aharsh environmental conditions [6]. Additionally, *A. baumannii* can develop resistance to numerous antimicrobial agents using different mechanisms [7]. It is well documented that one of the most important factors contributing to the high mortality of *A. baumannii* infections is the ability to acquire a wide variety of antibiotic resistance genes and rapidly develop multidrug resistance (MDR), extensive drug resistance (XDR) and even pan-drug resistance (PDR) [8]. Dissemination of MDR *A. baumannii* strains has significantly limited the choice of therapeutic options available for the treatment of infections caused by this bacterium and the associated poor clinical outcome [9]. 

According to previously published data, carbapenems are considered as the “last-line” antibiotic against infections caused by MDR *A. baumannii* strains in patients and healthcare workers [10]. Due to a severely limited range of alternative therapeutic options, unfortunately, recent reports described an increasing trend of multi-drug resistance in *A. baumannii* in many parts of the world, so that carbapenem resistant *A. baumannii* strains have emerged as a major public health concern [11], [12].

However, OXA carbapenemases are significantly inhibited by clavulanic acid, sulbactam and tazobactam [13]. Thus, increasing meropenem and ciprofloxacin susceptibility in *A. baumannii* by considering the potential inhibitory effect of tazobactam on OXA enzymes was examined in this study.

*Acinetobacter* species can acquire resistance against carbapenems by producing various carbapenemase enzymes, which are members of the molecular class A, B, and D β-lactamases. The class D carbapenemases, which consist of OXA-type β-lactamases (OXA) such as *bla_OXA-23-like_*, *bla_OXA-24-like_*, *bla_OXA-51-like_*, and *bla_OXA-58-like_*, are frequently detected in MDR *A. baumannii* strains [14]. Although clinical use of carbapenem agents in the treatment of infections has become well established, the use of this antibiotic alone must be limited due to concerns about the emergence and spread of resistant strains. Moreover, the high mortality rates of carbapenem-resistant *A. baumannii* infections highlight the importance of early prediction and appropriate control measures of this bacterium in health-care settings [15]. However, little information is available on whether different treatment regimens should be used for carbapenem-resistant *A. baumannii* infections. Given the lack of novel antimicrobials available in the clinical setting in Iran, we investigated the effects of meropenem and ciprofloxacin alone and in combination with tazobactam on *A. baumannii* isolated from burn patients, in the attempt to more effectively employ available antibiotics. The aim of this study was to evaluate the synergistic effect of different concentrations of tazobactam with ciprofloxacin and meropenem, and also to detect *bla_OXA-24-like_*, *bla_OXA-40-like_* and the *bla_OXA-58-like_* genes.

## Materials and methods

### Sample collection and bacterial strains 

The current study was carried out on 47 clinical isolates of *A. baumannii* obtained from patients admitted to Shahid Motahari Burns Hospital, Tehran, in a two-month period from June 2018 to August 2018. The study protocol was approved by the Ethics Committee of the National Institutes for Medical Research Development (IR NIMAD REC 1396 223), Tehran, Iran. Strains were identified by conventional biochemical and microbiological methods, e.g. oxidase, TSI, SIM, etc. In addition, to confirm *A. baumannii* identification, amplification and sequencing of intrinsic *bla_OXA-51-like_* genes were carried out using specific primers, as previously described [16]. All strains were stored in Tryptic Soy Broth (TSB; Merck, Germany) containing 20% glycerol at –80°C for further analysis.

### Antibiotic susceptibility testing

In-vitro susceptibility testing was performed using a panel of three antibiotics in the Kirby-Bauer disc diffusion method, according to the Clinical and Laboratory Standards Institute (CLSI 2018) [17] guidelines. The antimicrobial drugs tested included imipenem (10 µg), meropenem (10 µg) and ciprofloxacin (5 µg). 

*Escherichia coli* ATCC 25922 were used as a quality control strain in every test run. In this study, multi-drug resistance (MDR) was defined as non-sensitivity to ≥1 agent in ≥3 antimicrobial categories in CDC report [18]. 

### Minimum inhibitory concentration (MIC) assay

The minimum inhibitory concentrations (MIC) of meropenem and ciprofloxacin were determined alone and in combination with tazobactam against the *A. baumannii* isolates by a macro broth dilution according to the CLSI 2018 guideline [17]. Specifically, the following concentrations were used: meropenem: 256 µg/ml to 16 µg/ml; ciprofloxacin: 128 µg/ml to 16 µg/ml. All antimicrobials were purchased from Sigma-Aldrich (St. Louis, MO, USA).

### Synergic effect of tazobactam and antibiotics assay

The minimum inhibitory concentration of each strain against meropenem and ciprofloxacin with different concentrations of tazobactam was determined. 10 µg/ml, 30 µg/ml and 50 µg/ml tazobactam were used.

### Detection of carbapenem

DNA of the isolates was extracted using the boiling method as described previously [16]. The existence of class D carbapenemase genes (*bla_OXA-24-like_*, *bla_OXA-40-like_*, and *bla_OXA-58-like_*) was determined using PCR via specific primers (Table 1 (Tab. 1)). The PCR products were detected by agarose gel electrophoresis (1.5%), then they were stained with ethidium bromide and visualized under UV light (UVItec, Cambridge, UK). 

## Results

The results of the Kirby-Bauer disc diffusion test indicated that all of the tested isolates were resistant to meropenem, imipenem and ciprofloxacin. Therefore, all isolates were considered MDR and carbapenem-resistant *A. baumannii*. 

Table 2 (Tab. 2) shows the MICs (µg/mL) and the susceptibility ratios of the MDR and carbapenem-resistant *A. baumannii* isolates for meropenem and ciprofloxacin alone and in combination with tazobactam. The MICs exhibited manifold decreases between 10 µg/mL and 30 µg/mL with 50 µg/mL in the cases of combination use of ciprofloxacin and meropenem with tazobactam. In some cases, the results showed that more than one fold reduction in compare with 50 µg/mL and 10 µg/mL, although using tazobactam alone for *A. baumannii* had no inhibitory effect, and all isolates grew. 

**Figure 1:**
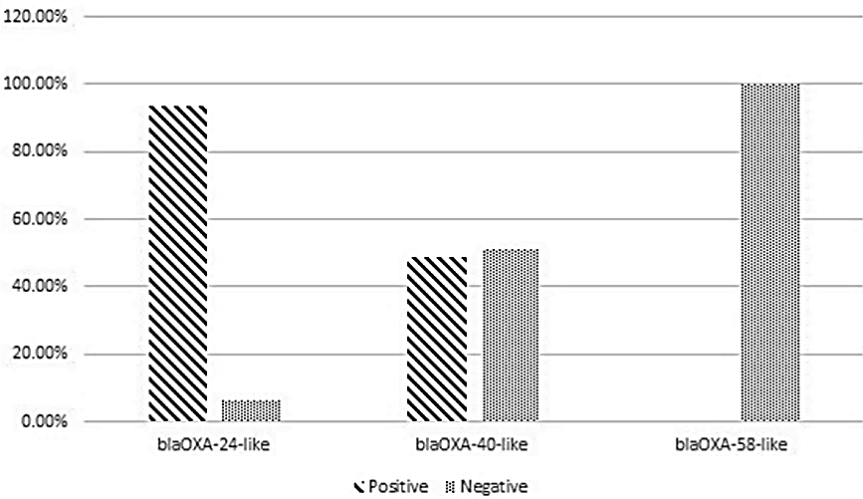
Frequency of resistance genes among *A. baumannii* isolated from burn patients hospitalized in Shahid Motahari Burns Hospital, Tehran

According to the results of the present study, *bla_OXA-24-like_* was the predominant carbapenemase gene (93.6%), followed by *bla_OXA-40-like_*, which was detected in 48.9% of isolates. None of the *A. baumannii* isolates harbored the *bla_OXA-58-like_* gene (Figure 1 (Fig. 1)). Furthermore, the co-existence of *bla_OXA-24-like_/bla_OXA-40-like_* was detected in 48.9% of *A. baumannii* isolates.

## Discussion

In recent decades, the emergence of MDR and carbapenem-resistant *A. baumannii* isolates with a high potential for acquiring resistance to various antibiotics has been described in health settings worldwide [12], [19]. Our results indicated that all *A. baumannii* isolates were MDR and carbapenem resistant. The high prevalence of MDR A. baumannii strains is in accordance with the findings reported by Farsiani et al. (97%) and Rynga et al. (85%) in Iran and India, respectively [20], [21]. The global spread of MDR clones in healthcare settings has raised a great deal of concern, because carbapenem agents are commonly the first choice in the treatment of *A. baumannii* infections [22], [23]. The high prevalence of MDR and carbapenem-resistant *A. baumannii* can be attributed to the indiscriminate use of antibiotics and poor implementation of measures.

The spread of these resistant strains has impeded the successful treatment of *A. baumannii* infections, thus necessitating alternative treatment approaches. Among the recommended approaches, the use of a combination of antibiotics is currently the preferred treatment strategy [24]. Combination therapy is principally used to avoid the development of antimicrobial resistance, treat polymicrobial infections, and decrease dose-dependent side effects. Moreover, it is also used to treat severe infectious diseases with high mortality rates, as a combination of antimicrobial agents provides a synergistic effect against the multi-drug-resistant isolates [25]. However, the absence of antagonistic interaction among antibiotics in cases of combination therapy has clinical importance; thus, many studies have emphasized the need to determine the interactive effects of antibiotic combinations in vitro [26]. It has been previously described that the combined administration of aminoglycoside and carbapenem agents, which are the most frequently used combination in the empiric treatment of Acinetobacter infections, generally demonstrates an in vitro synergistic effect [27]. 

The present study attempted to investigate the in vitro interactions between tazobactam and two antibiotics, meropenem and ciprofloxacin, as possible treatment options given carbapenem-resistant *A. baumannii* isolates from burn patients. Although sulbactam alone has verified antibacterial activity against *A. baumannii* and has intrinsic bactericidal activity against MDR *A. baumannii* as it inhibits the penicillin- binding proteins, there are no well-documented clinical practice guidelines for tazobactam and clavulanate [26]. Tazobactam has long been used in combination with ampicillin and piperacillin, and an additive effect against clinical isolates of *A. baumannii* was recently observed when tazobactam was combined with meropenem or colistin [28]. However, in this study, a significant reduction in MIC was observed for meropenem when combined with tazobactam. Moreover, the in vitro efficacy of ciprofloxacin/tazobactam combinations was evaluated against *A. baumannii* isolates. Our findings revealed a significant reduction in MIC when ciprofloxacin and meropenem were combined with tazobactam. These results are in accordance with data reported by several authors when sulbactam was combined with amikacin and ciprofloxacin [26], [29], [30]. 

Our finding is in accordance with the study by Rezaei et al. in 2018 in Isfahan that indicated *blaOXA-51-like* was present in all strains [31]. Moreover, Mohammadi et al. reported similar results among hospitalized patients with burn infection in 2016 in Iran [32]. Therefore, it is not surprising that Chen et al. in 2017 in China, Uwingabiye et al. in 2017 in Morocco, and Nowak et al. in 2017 in Greece, Italy, and Spain reported similar results in their investigations [33], [34], [35]. The percentage of *bla_OXA-24-like_* genes, which encode acquired carbapenemases, was 93.6% in the present study, followed by *bla_OXA-40-like_* with 48.9%. Furthermore, *bla_OXA-58-like_* was not detected in our study. Accordingly, in a study in Iran, the percentage of the *bla_OXA-24-like_* gene among tested isolates was 62.1% and the *bla_OXA-58-like_* was not detected among the isolates in that study [31]. In contrast to our results, Taherikalani et al., Salehi et al., and Sohrabi et al. reported the percentage of *bla_OXA-58-like_* to be 21.2%, 11.2%, and 3.2%, respectively [36], [12], [37]. Additionally, other studies in Turkey, China, Brazil, and France indicated the presence of the *bla_OXA-58-like_* gene in *A. baumannii* isolates [38], [39], [40], [41]. 

The results of the present study demonstrated that the co-existence of *bla_OXA-24-like_/bla_OXA-40-like_* in half of the *A. baumannii* isolates. In this regard, our results and those of others confirmed that the presence of multiple alleles of the *bla_OXA_* gene or a combination of them can be directly related to the reduction of the sensitivity or resistance to some antibiotics [42], [43].

## Conclusions

The results of this first study in Tehran demonstrate a high level of MDR and carbapenem-resistant *A. baumannii* isolates from burn patients. From a molecular standpoint, the existence of class D carbapenemase genes was established among a majority of the *A. baumannii* strains. Based on in vitro antimicrobial susceptibility in the current study, the MICs of tazobactam combined with meropenem or ciprofloxacin have been shown to be variable. Given the different mechanisms of antibiotic resistance in clinical isolates of *A. baumannii*, all results observed with a given combination is expected among *A. baumannii* strains. Furthermore, the data acquired from such *in vitro* conditions should be confirmed by reliable results from sufficiently controlled clinical trials. Because previous studies confirmed the inhibitory effect of tazobactam on OXA enzymes, the synergistic effect of tazobactam with ciprofloxacin and meropenem reflected in decreased MIC may be held responsible for inhibiting the identified OXA enzymes in the tested bacteria. In this study, also bacterial MIC was in antibiotic resistance range, so several mechanisms may be involved in the emergence of these resistances. Further investigation is necessary.

## Notes

### Competing interests

The authors declare that they have no competing interests.

### Funding 

The research reported in this publication was supported by the Elite Researcher Grant Committee under award number [IR NIMAD REC 1396 223] from the National Institutes for Medical Research Development (NIMAD), Tehran, Iran.

## Figures and Tables

**Table 1:**
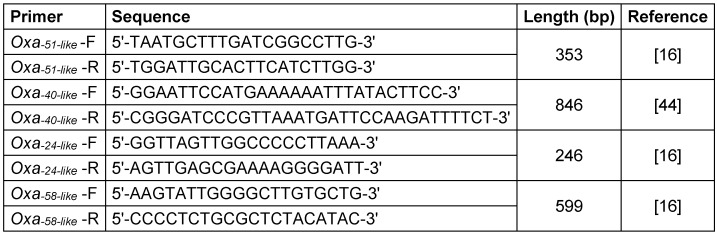
Oligonucleotide primers used in this study

**Table 2:**
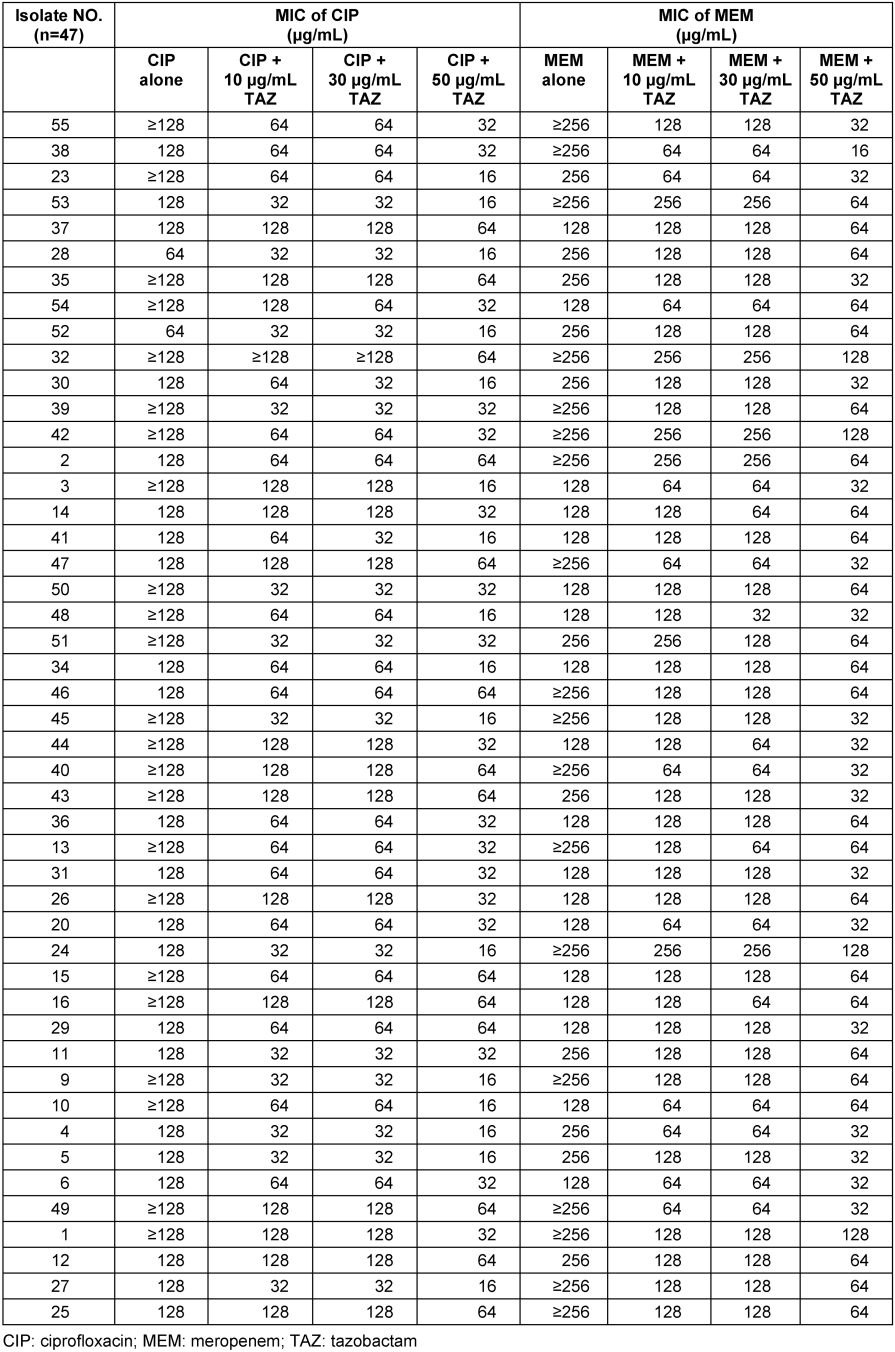
MIC intervals and susceptibility ratios against *A. baumannii* isolated from burn patients hospitalized in Shahid Motahari Burns Hospital, Tehran
